# Computed tomography values of pericardial effusion may predict chylopericardium: a case report

**DOI:** 10.1186/s12872-023-03112-2

**Published:** 2023-02-10

**Authors:** Marohito Nakata, Naoko Yokota, Tsuneaki Kenzaka

**Affiliations:** 1 Department of Cardiology, Naha City Hospital, Naha, Okinawa Japan; 2 Division of Community Medicine and Career Development, Kobe University Graduate School of Medicine, 2-1-5, Arata-cho, Hyogo-ku, Kobe, Hyogo 652-0032 Japan

**Keywords:** Idiopathic chylopericardium, Hounsfield units, Computed tomography, Chylous fluid, Prediction, Pericardial effusion, Pericardiocentesis

## Abstract

**Background:**

Idiopathic chylopericardium is a rare disease characterized by filling of the pericardial cavity with chylous fluid and has no evident cause. Secondary chylopericardium usually results from injury or damage to the thoracic duct. The most common causes of secondary chylopericardium are trauma, thoracic or cardiac surgery, and congenital lymphangiomatosis. Conservative or surgical treatment can be pursued; however, surgical treatment is required if conservative treatment is unsuccessful. Pericardiocentesis plays a crucial role in the definitive diagnosis of chylopericardium. However, although a serious complication, its occurrence is infrequent. Non-invasive methods, such as computed tomography (CT), could be useful in predicting the color or characteristics of pericardial effusion.

**Case presentation:**

A 37-year-old Japanese woman presented to our hospital with a cough that persisted for 1 week. Echocardiography revealed pericardial effusion, which was diagnosed as acute pericarditis and treated with loxoprofen. However, pericardial effusion increased, and the patient presented to the emergency room with cardiac tamponade 1 month later. Pericardiocentesis was performed, which confirmed that the pericardial effusion was chylopericardium. Lymphatic scintigraphy did not show any connection between the thoracic duct and pericardial cavity, and the patient was diagnosed with idiopathic chylopericardium. The patient underwent continuous drainage for 11 days. After completion of cardiac drainage, the patient was discharged from the hospital without any exacerbation. The CT attenuation value of the pericardial fluid was 11.00 Hounsfield units (HU). Compared with the other causes of pericardial effusions encountered at our hospital, the HU on CT scan of pericardial effusion was low in our study and similar to the values on CT scan of chylous ascites reported in previous studies.

**Conclusions:**

Although idiopathic chylopericardium is rare, it should be considered an important cause of pericardial effusion. Pericardiocentesis is necessary for definitive diagnosis; however, the CT findings of pericardial effusion may help predict the presence of chylous fluid.

## Background

Idiopathic chylopericardium is a rare disease characterized by the retention of chylous fluid in the pericardial cavity, and only 104 cases have been reported in 65 years since 1950 [1]. The time from onset of symptoms to diagnosis demonstrates a wide range, extending from a few hours to several years [1]; therefore, it is important to gain further insight into this disease. Treatment can be conservative or surgical; however, the frequency of patients requiring surgical treatment after the failure of conservative therapy is as high as 58.0–66.7% [1, 2]. Pericardiocentesis is essential for the definitive diagnosis of chylopericardium. Although the frequency is low (< 2%), it can cause serious complications [3, 4]. If the characteristics of pericardial effusion can be predicted via non-invasive methods, such as computed tomography (CT), before pericardiocentesis, conservative treatment can be attempted first, which may be useful in daily practice. In this study, we report an extremely rare case of idiopathic chylopericardium and compared the Hounsfield units (HU) on CT scan of this case with those of other cases of pericardial effusion encountered at our hospital.

## Case presentation

### Patient characteristics

**Fig. 1:**
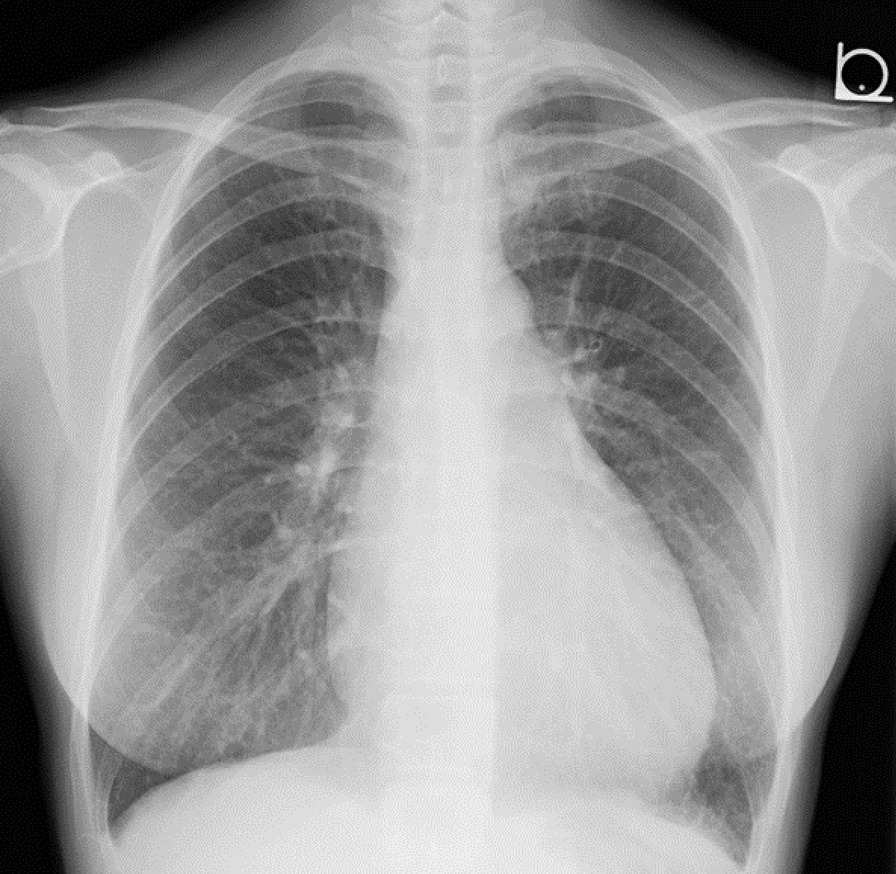
Chest radiograph acquired at the initial visit. The chest radiograph shows cardiomegaly. The cardiothoracic ratio is 52.4%

**Fig. 2:**
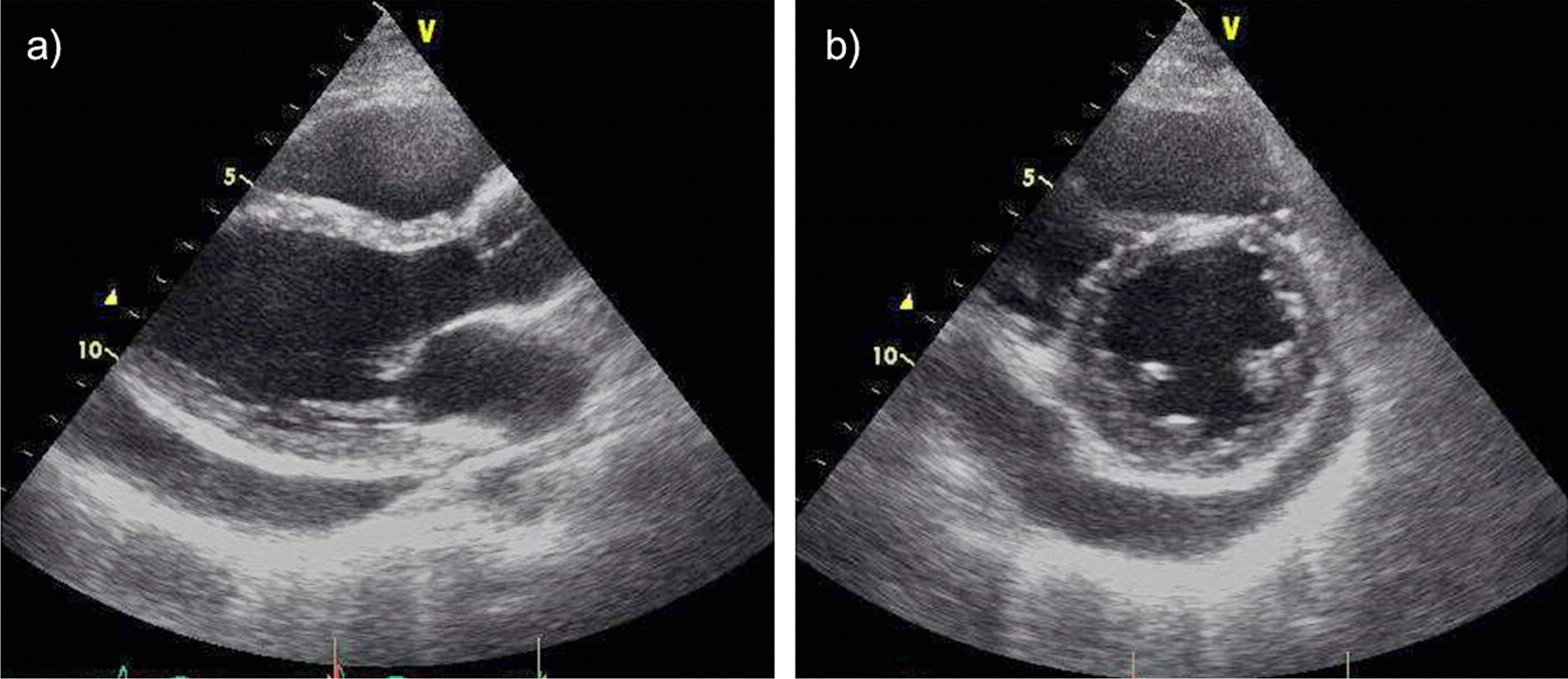
Transthoracic echocardiogram at the initial visit. **a** Parasternal left border long-axis tomogram, **b** parasternal left border short-axis tomogram. Echocardiography showing pericardial effusion

A 37-year-old Japanese woman presented to our hospital with a complaint of a cough persisting for 1 week. She had a history of bronchial asthma but was not currently undergoing regular follow-up. She was referred to our hospital because of a suspected asthma attack after visiting her family doctor. Her blood pressure was 114/74 mmHg at the initial visit. Chest radiography revealed cardiomegaly with a cardiothoracic ratio (CTR) of 52.4% (Fig. 1). Echocardiography revealed pericardial effusion (Fig. 2). Hypothyroidism and systemic lupus erythematosus (SLE) were considered possible differential diagnoses; however, the thyroid stimulating hormone (TSH) and thyroxine (T4) levels were normal, and antinuclear antibody (ANA) was absent. Therefore, hypothyroidism and SLE were ruled out (Table 1). Acute pericarditis was suspected; thus, loxoprofen sodium hydrate (60 mg, three times a day for 7 days) was prescribed. A chest radiograph acquired 1 week after initiating drug therapy confirmed that there was no worsening of cardiac enlargement. However, the patient was admitted to our hospital approximately 1 month after the initial diagnosis due to chest pain and dyspnea.

**Table 1:** Laboratory findings at the initial visit

Parameter	Recorded value	Standard value
White blood cell count	6300/µL	3300–8600/µL
Hemoglobin	13.7 g/dL	11.5–15.0 g/dL
Platelet count	26.5 × 10^4^/µL	15–35 × 10^3^/µL
C-reactive protein	0.04 mg/dL	≤ 0.14 mg/dL
Total protein	7.0 g/dL	6.6–8.1 g/dL
Albumin	4.1 g/dL	4.1–5.1 g/dL
Aspartate aminotransferase	12 U/L	13–30 U/L
Alanine aminotransferase	11 U/L	7–23 U/L
Lactase dehydrogenase	151 U/L	124–222 U/L
Blood urea nitrogen	10.2 mg/dL	8–20 mg/dL
Creatinine	0.58 mg/dL	0.46–0.79 mg/dL
Sodium	140 mEq/L	138–145 mEq/L
Potassium	4.1 mEq/L	3.6–4.8 mEq/L
Chloride	106 mEq/L	101–108 mEq/L
Glucose	110 mg/dL	75–110 mg/dL
Thyroid stimulating hormone	1.430 μIU/L	0.34–4.22 μIU/L
Free T4	1.35 ng/dL	0.77–1.74 ng/dL
Myeloperoxidase (MPO) antineutrophil cytoplasmic antibody	(-)	
Antinuclear antibody	< 40 times	
Anti-double stranded DNA IgG antibody	(-)	
Erythrocyte sedimentation rate 1-h	10 mm	< 15 mm
2-h	28 mm	< 40 mm
Total cholesterol	161 mg/dL	142–248 mg/dL
Triglyceride	56 mg/dL	30–117 mg/dL
High density lipoprotein cholesterol	59 mg/dL	48–103 mg/dL
Low density lipoprotein cholesterol	89 mg/dL	65–163 mg/dL
Hemoglobin A1c	5.3%	4.9–6.0%

### Investigations

On admission, her vital signs were as follows: blood pressure, 86/52 mmHg; heart rate, 73 beats/min; respiratory rate, 20 breaths/min; body temperature, 36.6 °C; and partial pressure of oxygen (SpO_2_), 98% (ambient air). Chest radiography revealed worsening of the cardiac enlargement (CTR, 62.2%), and chest CT revealed significant pericardial effusion. The CT attenuation value of the pericardial fluid was 11.00 HU.

### Differential diagnosis

**Fig. 3:**
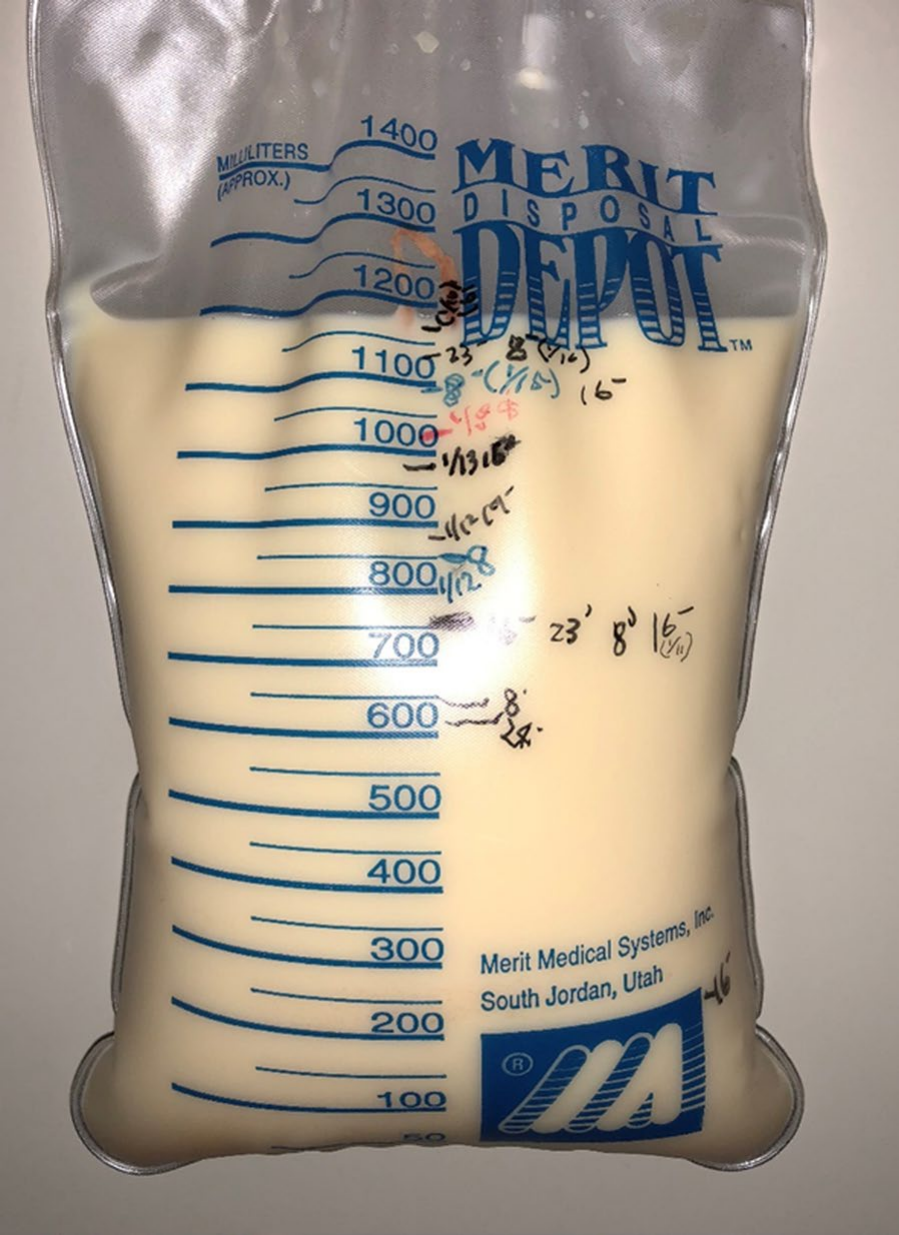
Pericardial fluid in the drainage bag. Pericardial effusion fluid with milky white appearance

**Fig. 4:**
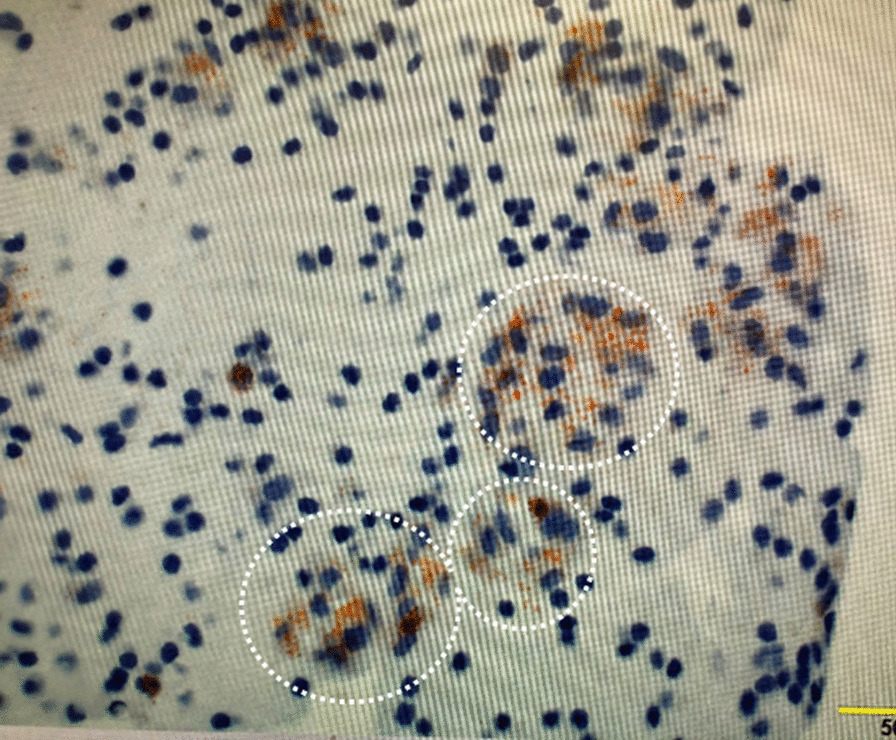
Sudan III staining of the pericardial effusion fluid. The figure depicts positive staining. Triglyceride in pericardial fluid is dyed orange (white circles)

Since the patient was experiencing chest pain and dyspnea and had low blood pressure, we diagnosed her with cardiac tamponade and performed pericardiocentesis. The pericardial effusion was milky white (Fig. 3). Analysis of the pericardial effusion (Table 2) revealed a triglyceride (TG) level of 3220 mg/dL and a total cholesterol (T-CHO)/TG ratio of < 1. Cytological examination revealed numerous lymphocytes. Fat staining and Sudan III staining showed positive findings (Fig. 4). Bacteria were not detected in the cardiac effusion culture, and tuberculosis was not identified. Thus, the patient was diagnosed with chylopericardium.

**Table 2:** Analysis of the pericardial effusion fluid

Parameter	Recorded value
Total protein	5 g/dL
Albumin	4 g/dL
Cell count	**939**
Total cholesterol	**87 mg/dL**
Triglyceride	**3220 mg/dL**

Bold values are outliers

**Fig. 5:**
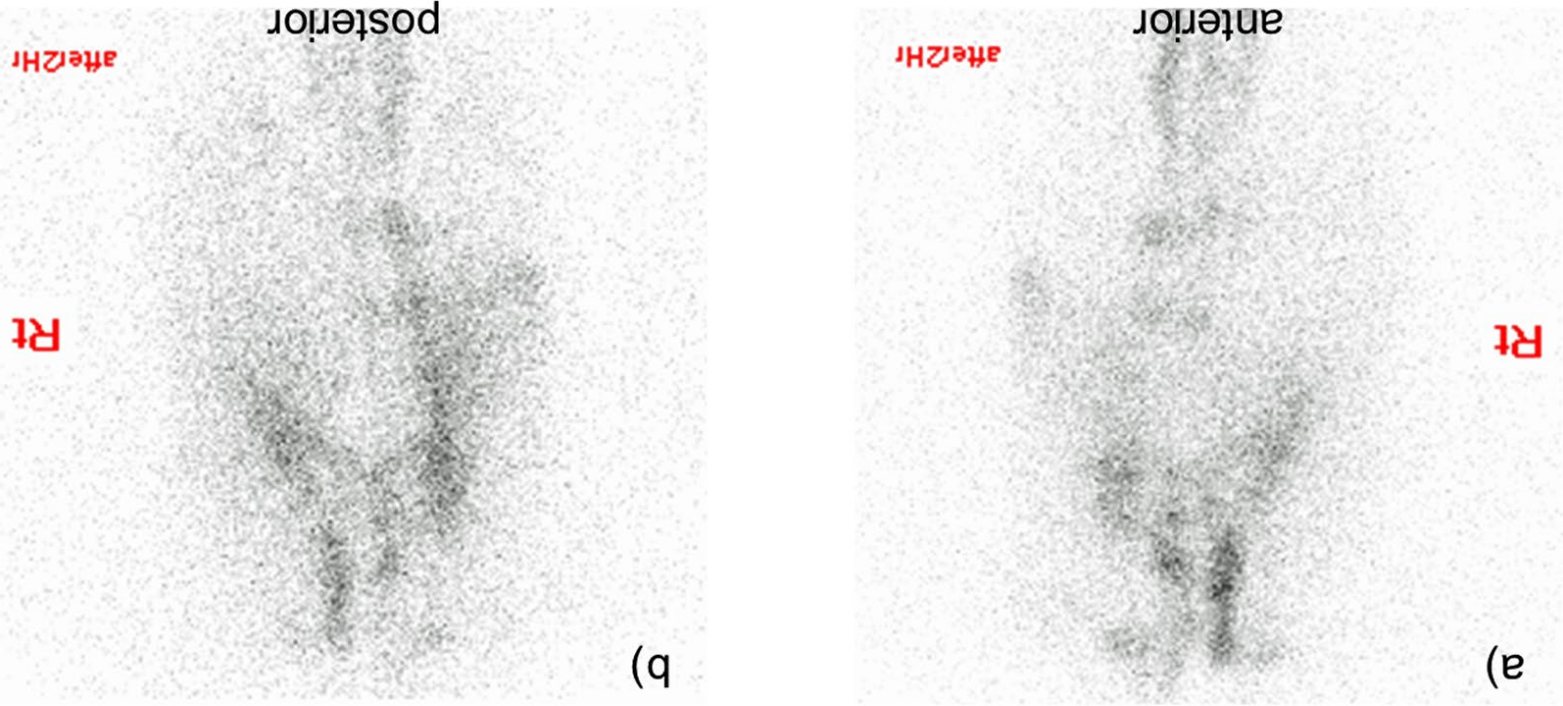
Lymphoscintigraphy. **a** Anterior view, **b** posterior view. The images shown were taken 2 h after subcutaneous injection with Technetium-99m sulfur colloid. They do not reveal any connection between the thoracic duct and the pericardial cavity

Lymphoscintigraphy did not reveal any connection between the thoracic duct and pericardial cavity (Fig. 5); therefore, the patient was diagnosed with idiopathic chylopericardium.

### Outcome and follow-up

A drainage tube was placed after pericardiocentesis, and continuous drainage was performed for 11 days. Chest pain and dyspnea improved after drainage. Pericardial effusion increased temporarily after the removal of the drainage tube; however, it decreased spontaneously thereafter. The patient was discharged on the 21st day of hospitalization without recurrence of chest pain or dyspnea. Four years after the onset of the disease, no recurrence has been reported thus far.

## Discussion

Herein, we present a case of idiopathic chylopericardium. Although an extremely rare cause of pericardial effusion [1], it should be considered a differential diagnosis. To the best of our knowledge, no previous reports have measured the HU on CT scan of chylous pericardial effusion in adults. The low HU on CT scan in the present study was similar to that reported for chylous ascites [5].

The most common causes of pericardial effusion are acute pericarditis, autoimmune diseases, post-myocardial infarction or cardiac surgery, malignant tumors, mediastinal radiation therapy, and renal failure with uremia [6]. Acute pericarditis was suspected in this case, and treatment was initiated; however, the patient’s condition deteriorated 1 month later.

Chylopericardium may be primary or secondary. Primary chylopericardium is a rare disease, with only approximately 100 cases reported in the past [1]. Secondary chylopericardium occurs more frequently than primary chylopericardium because of the postoperative complications from cardiac surgery, trauma, thrombosis in the jugular vein, infection, radiotherapy, mediastinal tumor, lymphoma, acute necrotizing pancreatitis, and malignant tumors [7]. Four mechanisms have been reported as causes of idiopathic primary chylopericardium [1]: (1) failure of the lymphatic valves in the branches connecting the thoracic duct and pericardium lymphatic vessels; (2) increased pressure in the thoracic duct, which can occur in lymphangiectasia; (3) abnormal communication between the lymphatic vessels and the pericardial lymphatics resulting in chylous reflux; and (4) congenital malformation. In the present case, lymphoscintigraphy showed no evidence of lymphatic dilation, abnormal pericardial lymphatic connection, or congenital malformation; thus, primary chylopericardium was diagnosed based on the absence of secondary causes.

Pericardiocentesis is essential for the definitive diagnosis of chylopericardium. The following characteristics were noted in addition to the milky, opaque, or opalescent pericardial fluid: TG level > 500 mg/dl, T-CHO/TG ratio < 1, no abnormalities in culture or cytology, and Sudan III staining revealing significant lymphocytes and adipocytes [8]. Echocardiography, CT, and electrocardiography findings were not characteristic of idiopathic chylopericardium.

**Fig. 6:**
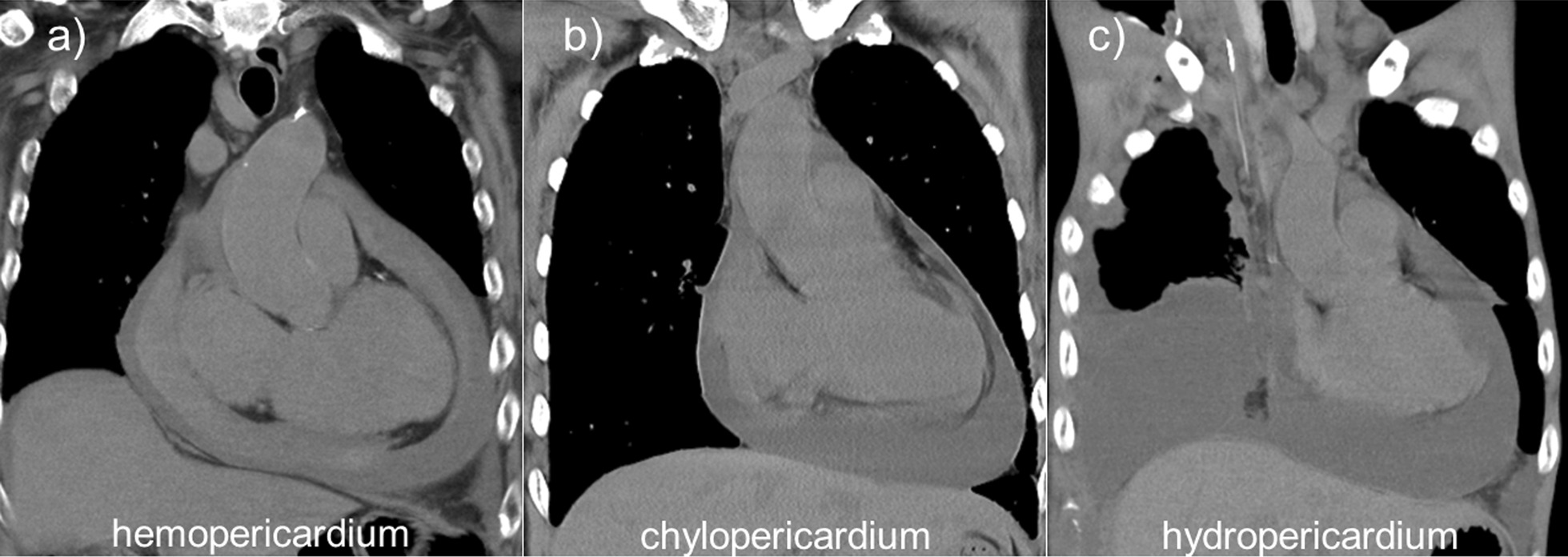
Computed tomography (CT) images comparing **a** hemopericardium, **b** chylopericardium, and **c** hydropericardium. The mean Hounsfield units (HU) on the CT scan of hemopericardium (22 cases) and hydropericardium (15 cases) were 22.32 and 15.39 HU, respectively. For chylopericardium (1 case, presented in the current report), it was 11.00 HU

Therefore, we investigated the possibility of predicting the color of the pericardial effusion fluid and determining whether it is chyle by obtaining the HU on CT scan. We measured the CT attenuation values of 44 consecutive patients who underwent pericardiocentesis between January 1, 2014, and April 31, 2021 (January 1, 2014–March 31, 2017; Aquilion CX, Canon, Tokyo, Japan, and Revolution CT, GE Healthcare, Chicago, IL, USA, since April 1, 2017) (Fig. 6). CT images were acquired within 1 month of pericardiocentesis in all cases. The HU on CT scan of the anterior, posterior, and lateral sides of the pericardial cavity were measured, and the mean value was calculated [9]. We analyzed 38 cases, excluding six cases in which the color of the collected pericardial effusion fluid was not described. Based on the appearance of the fluid, the conditions were categorized as hemopericardium, hydropericardium, chylopericardium, or purulent pericardium. The mean CT attenuation value of hemopericardium (22 cases), hydropericardium (15 cases), and chylopericardium (1 case, presented in the current report) was 22.32, 15.39, and 11.00 HU, respectively. No cases of purulent pericardium were reported. The mean CT attenuation value of chylopericardium was lower than that of hemopericardium or hydropericardium. There are no reports on the HU on CT scan of the pericardial fluid in chylopericardium; however, the HU on CT scan of chylous ascites has been reported to be as low as 7–10 HU [5]. Since the HU on CT scan of blood is high and that of fat is low, it is assumed that the HU increases when the pericardial effusion contains blood and decreases when the effusion contains fat. The HU on CT scan was low in the present case, similar to that reported in previous cases of chylous ascites. To the best of our knowledge, no previous study has reported on the HU on CT scan of chylopericardium in adults, and only one study has reported a case of a 7-month-old female infant [10], in whom the HU on CT scan was + 6 to – 6 HU, similar to our results. The HU on CT scan of chylopericardium could be as low as that of chylous ascites. Patients with low HU of pericardial effusion may have chylopericardium.

Among 102 patients diagnosed with idiopathic chylopericardium, 62 received conservative treatment, whereas 36 (58.0%) required surgery [1]. Overall, 71 patients (71.2%) underwent surgery [1]. Thus, the probability of improvement with conservative treatment is low. Pericardial window opening (48.1%) and thoracic duct ligation (44.2%) were the most common surgical procedures [1]. In the present case, chest pain and dyspnea appeared approximately 1 month after the initial consultation, which prompted pericardiocentesis and led to the diagnosis. Since a moderate amount of pericardial effusion was observed at the initial examination, an early diagnosis could have been made if pericardiocentesis had been performed earlier. Pericardiocentesis is associated with several complications. Although the frequency of serious complications is low (< 2%), myocardial puncture or muscle injury, vascular injury, pneumothorax, and arrhythmia can cause serious conditions, making patients hesitant to undergo pericardiocentesis [3, 4]. This was especially true for young patients in the present study. The HU on CT scan may aid in predicting the nature of the pericardial effusion fluid in such cases.

## Conclusion

Although rare, idiopathic chylopericardium should be considered a cause of pericardial effusion. Pericardiocentesis is necessary for definitive diagnosis; however, CT findings of pericardial fluid may indicate possible accumulation of chylous fluid.

## Data Availability

All data generated or analyzed during this study are included in this published article.

## Abbreviations

CT: Computed tomography
HU: Hounsfield units
CTR: Cardiothoracic ratio
SLE: Systemic lupus erythematosus
TSH: Thyroid stimulating hormone
ANA: Antinuclear antibody
TG: Triglyceride
CHO/TG: Total cholesterol and triglyceride ratio
SpO_2_: Partial pressure of oxygen
